# Significance of RNA N6-Methyladenosine Regulators in the Diagnosis and Subtype Classification of Childhood Asthma Using the Gene Expression Omnibus Database

**DOI:** 10.3389/fgene.2021.634162

**Published:** 2021-03-02

**Authors:** Bing Dai, Feifei Sun, Xuxu Cai, Chunlu Li, Henan Liu, Yunxiao Shang

**Affiliations:** ^1^Department of Pediatrics, Shengjing Hospital of China Medical University, Shenyang, China; ^2^Department of Ultrasound, Shengjing Hospital of China Medical University, Shenyang, China; ^3^Department of Ophthalmology, Shengjing Hospital of China Medical University, Shenyang, China

**Keywords:** childhood asthma, m6A RNA methylation regulators, m6A-related genes, diagnostic biomarkers, consensus clustering

## Abstract

RNA N6-methyladenosine (m6A) regulators play important roles in a variety of biological functions. Nonetheless, the roles of m6A regulators in childhood asthma remain unknown. In this study, 11 significant m6A regulators were selected using difference analysis between non-asthmatic and asthmatic patients from the Gene Expression Omnibus GSE40888 dataset. The random forest model was used to screen five candidate m6A regulators (fragile X mental retardation 1, KIAA1429, Wilm’s tumor 1-associated protein, YTH domain-containing 2, and zinc finger CCCH domain-containing protein 13) to predict the risk of childhood asthma. A nomogram model was established based on the five candidate m6A regulators. Decision curve analysis indicated that patients could benefit from the nomogram model. The consensus clustering method was performed to differentiate children with asthma into two m6A patterns (clusterA and clusterB) based on the selected significant m6A regulators. Principal component analysis algorithms were constructed to calculate the m6A score for each sample to quantify the m6A patterns. The patients in clusterB had higher m6A scores than those in clusterA. Furthermore, we found that the patients in clusterA were linked to helper T cell type 1 (Th1)-dominant immunity while those in clusterB were linked to Th2-dominant immunity. In summary, m6A regulators play nonnegligible roles in the occurrence of childhood asthma. Our investigation of m6A patterns may be able to guide future immunotherapy strategies for childhood asthma.

## Introduction

Bronchial asthma, the most common chronic respiratory disease in children, is characterized by airway inflammation, airway hyperreactivity, and airway remodeling (Troy et al., 2016). Approximately 50% of preschoolers have had at least one wheeze (Martinez et al., 1995), but only a small number of wheeze children later develop asthma. At present, there is no “gold standard” for the diagnosis of asthma in children under 6years old. Therefore, early identification of children at high risk of developing asthma among young wheezing children is critical. Given extensive developments in asthma research, it is now believed that asthma is a complex and heterogeneous disease related to genetic changes (Chen et al., 2016). Therefore, early screening of high-risk children from a genetic standpoint and effective prevention will have a profound impact on the control of asthma prevalence.

More than 100 different types of RNA modification have been found in eukaryotes, including N6-methyladenosine (m6A), N1-methyladenosine, 5-methylcytidine (Dunin-Horkawicz et al., 2006; Roundtree et al., 2017). Among them, m6A has the highest modified abundance. M6A modification is a methylated modification formed by methyltransferase complex (MTC) methylation of the sixth position N of adenine on mRNA (Fu et al., 2014; Deng et al., 2018). M6A is an important epigenetic modification that requires multiple regulatory proteins encoded by writers, erasers, and readers to work together (Yang et al., 2018). The MTC is involved in the production of m6A modification while de-methylated transferase (FTO and ALKBH5) can remove m6A. Together, they maintain the dynamic balance between methylation and non-methylation of mRNA in the cell. Readers are a class of RNA binding proteins that can recognize the sites of m6A modification, bind to m6A, and participate in the regulation of mRNA metabolism along with the MTC and de-methylated transferase (Tong et al., 2018b; Chen et al., 2019). M6A methylation has important effects on the splicing process, stability, translation efficiency, and nuclear retention of mRNA (Dai et al., 2018). Recently, numerous studies have shown that m6A modification plays an important role in the occurrence and development of tumors by affecting the expression of tumor-related genes (Wang et al., 2020a,b; Zhang et al., 2020a). However, the roles of m6A regulators in childhood asthma remain unknown.

In this study, we comprehensively evaluated the functions of m6A regulators in the diagnosis and subtype classification of childhood asthma based on the GSE40888 dataset from the Gene Expression Omnibus (GEO) database. We established a gene model for predicting asthma susceptibility based on five candidate m6A regulators [fragile X mental retardation 1 (FMR1), KIAA1429, Wilm’s tumor 1-associated protein (WTAP), YTH domain-containing 2 (YTHDC2), and zinc finger CCCH domain-containing protein 13 (ZC3H13)] and found that patients could obtain a good clinical benefit based on the model. In addition, we revealed two distinct m6A patterns that were highly consistent with helper T cell type 1 (Th1)-dominant immunity and Th2-dominant immunity, suggesting that m6A patterns may be used to distinguish allergic asthma from non-allergic asthma and guide subsequent treatment.

## Materials and Methods

### Data Acquisition

The GSE40888 dataset containing 40 non-asthmatic and 65 asthmatic patients was obtained from the GEO database.^1^ We extracted a total of 21 m6A regulators from the dataset by identifying significant m6A regulators using difference analysis between non-asthmatic and asthmatic patients. These regulators consisted of eight writers (METTL3, ZC3H13, METTL14, RBM15B, CBLL1, WTAP, RBM15, and KIAA1429), two erasers (FTO and ALKBH5), and 11 readers (YTHDC1, YTHDC2, ELAVL1, YTHDF1, LRPPRC, YTHDF2, FMR1, YTHDF3, HNRNPC, HNRNPA2B1, and IGF2BP1).

### Construction of a Random Forest Model and Support Vector Machine Model

Random forest (RF) and support vector machine (SVM) model was constructed as a training model to predict the occurrence of childhood asthma. “Reverse cumulative distribution of residual,” “Boxplots of residual” and receiver operating characteristic (ROC) curve was plotted to evaluate the model. RF is a constituent supervised learning method that can be considered as an extension of a decision tree. In our research, “RandomForest” package in R statistical software (The R Foundation, Vienna, Austria) was used to establish an RF model to select candidate m6A regulators among the 21 m6A regulators to predict the occurrence of childhood asthma. In our research, ntrees and mtry were, respectively, set at 100 and 3. We then analyzed the importance of the 21 m6A regulators and selected the appropriate important m6A regulators through 10 fold cross-validation. The *Y*-axis of the 10-fold cross-validation curve corresponds to the accuracy of the model when selecting different numbers of m6A regulators. SVM is a supervised machine learning algorithm based on the structural risk minimization principle from statistical learning theory. In our research, every data point was plotted as a dot in n-dimensional spaces (where *n* is the number of the m6A regulators). Then, we find an optimal hyperplane that differentiates the two classes (non-asthma and asthma) very well (Bao et al., 2020).

### Construction of a Nomogram Model

We then constructed a nomogram model based on the selected candidate m6A regulators using the “rms” package in R to predict the prevalence of childhood asthma patients. The calibration curve was used to evaluate the consistency of our predicted values against reality. Decision curve analysis (DCA) was performed, and a clinical impact curve was plotted to assess whether decisions based on the model were beneficial to the patient (Iasonos et al., 2008).

### Identification of Molecular Subtypes Based on the Significant m6A Regulators

Consensus clustering is an algorithm that is used to identify each member and its subgroup number and verify clustering rationality based on resampling. The consensus clustering method was performed to identify distinct m6A patterns based on the significant m6A regulators using the “ConsensusClusterPlus” package in R (Wilkerson and Hayes, 2010).

### Identification of Differentially Expressed Genes Between Distinct m6A Patterns and Gene Ontology Functional Enrichment Analysis

The “limma” package in R was used to screen for differentially expressed genes (DEGs) between distinct m6A patterns. A *p* < 0.01 was selected as the screening criterion. GO functional enrichment analysis was applied to understand the possible mechanism of the DEGs involved in childhood asthma using the “clusterProfiler” package in R software, and the results were visualized with an enrichment circle diagram (Denny et al., 2018).

### Estimation of the m6A Gene Signature

To quantify the m6A patterns, we utilized principal component analysis (PCA) algorithms to calculate the m6A score for each sample. First, PCA was conducted to distinguish the m6A patterns. Then, the m6A score was calculated according to the following formula: m6A score = PC1_i_, where PC1 represents principal component 1, and *i* represents DEG expression (Zhang et al., 2020a).

### Estimation of Immune Cell Infiltration

Single sample gene set enrichment analysis (ssGSEA) was used to evaluate the abundance of immune cells in asthmatic samples. First, ssGSEA was used to sequence the gene expression levels in the samples to obtain their rank. Next, we searched for these genes in the input data set, after which the expression levels of these genes were summed. Based on the above evaluation, we obtained the abundance of immune cells in each sample (Zhang et al., 2020b).

### Statistical Analysis

Linear regression analyses were utilized to explore the correlation between writers and erasers. Kruskal-Wallis tests were used to compare differences between groups. All parametric analyses were based on two-tailed tests, the statistical significance for which was set at *p* < 0.05. All statistical analyses were performed using R version 4.0.0.

## Results

### Landscape of the 21 m6A Regulators in Childhood Asthma

**Figure 1:**
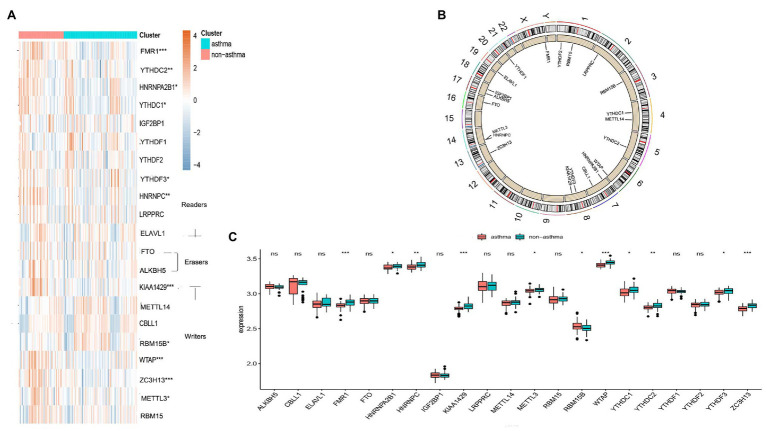
Landscape of the 21 RNA N6-methyladenosine (m6A) regulators in childhood asthma. **(A)** Expression heat map of the 21 m6A regulators in non-asthmatic and asthmatic patients. **(B)** Differential expression histogram of the 21 m6A regulators identified between non-asthmatic and asthmatic patients. **(C)** Chromosomal positions of the 21 m6A regulators. ^*^p < 0.05, ^**^p < 0.01, and ^***^p < 0.001.

The “limma” package in R was utilized to analyze the differential expression levels of 21 m6A regulators between non-asthmatic and asthmatic patients. Eleven significant m6A regulators (YTHDC1, HNRNPC, YTHDC2, FMR1, YTHDF3, HNRNPA2B1, KIAA1429, METTL3, WTAP, RBM15B, and ZC3H13) were screened and visualized using a heat map and histogram. We found that RBM15B was overexpressed in asthmatic patients while the other significant m6A regulators displayed decreased expression in asthma patients compared to non-asthmatic patients (Figures 1A,C). The chromosomal positions of the 21 m6A regulators were visualized using the “RCircos” package (Figure 1B).

### Correlation Between Writers and Erasers in Childhood Asthma

**Figure 2:**
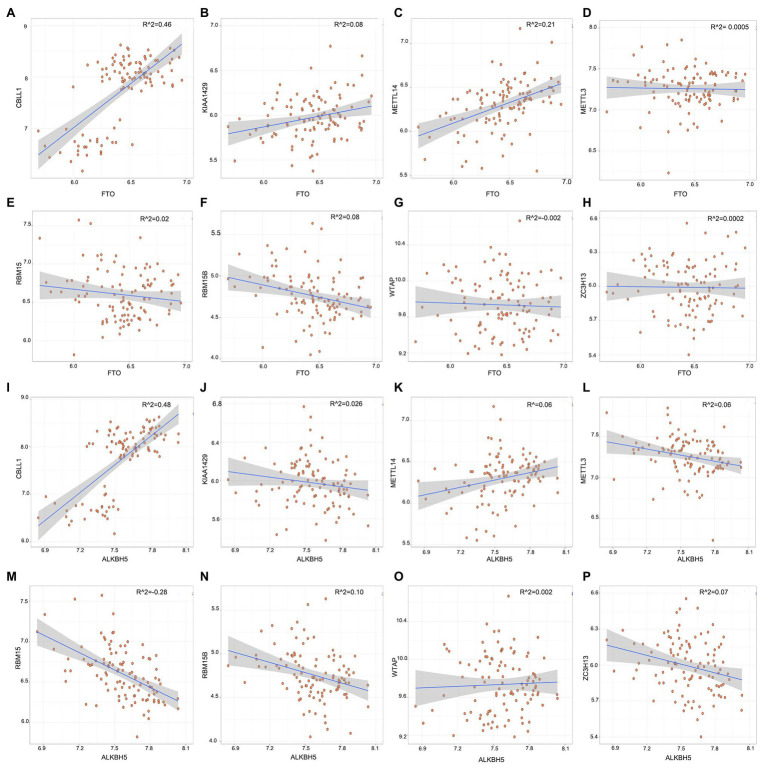
Correlation between writers and erasers in childhood asthma **(A-P)**. Writer genes: Wilm’s tumor 1-associated protein, RBM15, RBM15B, METTL14, METTL3, KIAA1429, CBLL1, and ZC3H1; eraser genes: ALKBH5 and FTO.

To explore whether high writer gene expression levels in childhood asthma exhibit low eraser gene expression levels, we utilized linear regression analyses were utilized to explore the correlation between writers and erasers. We found that the expression levels of CBLL1 and METTL14 in asthmatic patients had a high positive correlation with FTO. Asthmatic patients with high expression levels of RBM15B displayed low expression levels of ALKBH5 while high CBLL1 expression showed a positive correlation with ALKBH5. The other writers had no significant correlations with erasers (FTO and ALKBH5; Figure 2). Therefore, we demonstrated that various writers and erasers have different correlations with each other.

### Construction of the RF Model and SVM Model

**Figure 3:**
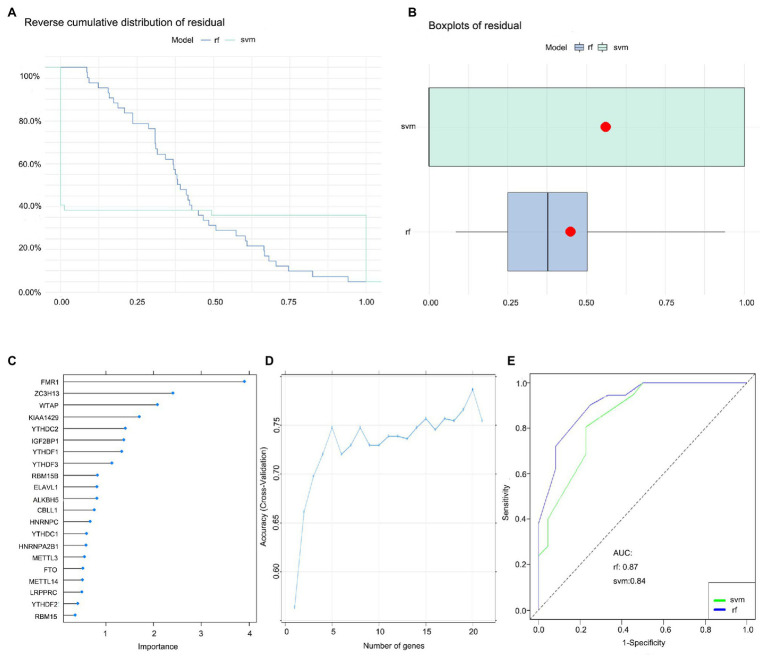
Random forest (RF) model construction. **(A)** Reverse cumulative distribution of residual was plotted to show the residual distribution of RF and support vector machine (SVM) model. **(B)** Boxplots of residual was plotted to show the residual distribution of RF and SVM model. **(C)** The importance of the 21 RNA N6-methyladenosine regulators based on the RF model. **(D)** Ten-fold cross-validation curve to assess the quality of childhood asthma prediction in the RF model. **(E)** ROC curves indicated the accuracy of the RF and SVM model.

We established an RF and SVM model to select candidate m6A regulators from the 21 m6A regulators to predict the occurrence of childhood asthma. Both “Reverse cumulative distribution of residual” (Figure 3A) and “Boxplots of residual” (Figure 3B) revealed that the RF model has minimal residuals. Most of the samples in the model have relatively small residuals, indicating that the model is better. Thus, the RF model was considered as the best model to predict the occurrence of childhood asthma. We visualized the 21 m6A regulators after ranking these genes according to their importance (Figure 3C). Ten-fold cross-validation curve indicated that the RF model has the highest accuracy when selecting the top 20 m6A regulators. However, the number of m6A regulators was in the top five (FMR1, KIAA1429, WTAP, YTHDC2, and ZC3H13) was selected as the candidate genes (Figure 3D). Finally, the ROC curve was plotted to evaluate the model and the AUC value of the ROC curve also indicated that the RF model has higher accuracy than SVM model (Figure 3E).

### Construction of the Nomogram Model

**Figure 4:**
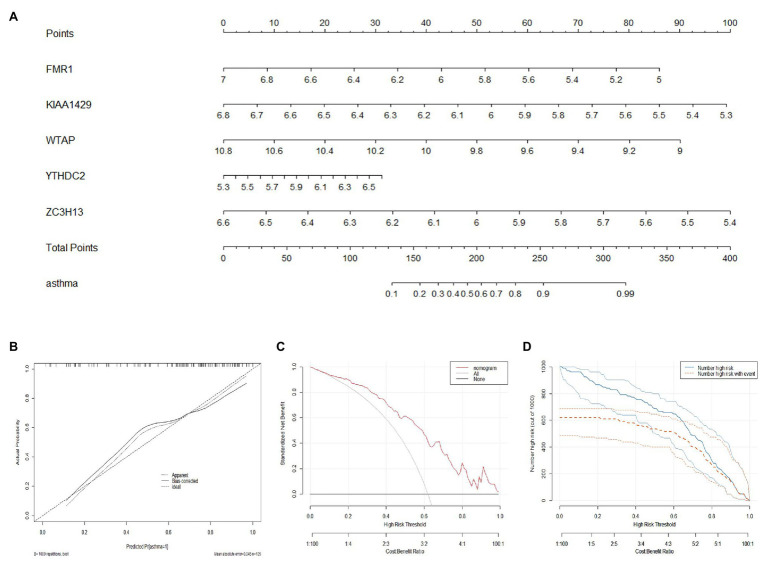
Construction of the nomogram model. **(A)** Construction of the nomogram model based on the five candidate RNA N6-methyladenosine regulators. **(B)** Predictive ability of the nomogram model as revealed by the calibration curve. **(C)** Decisions based on the nomogram model may benefit childhood asthma patients. **(D)** Clinical impact of the nomogram model as assessed by the clinical impact curve.

A nomogram model based on the five candidate m6A regulators was constructed using the “rms” package in R to predict the prevalence of childhood asthma patients (Figure 4A). Calibration curves revealed that the predictivity of the nomogram model was accurate (Figure 4B). The red line in the DCA curve remained above the gray and black lines from 0 to 1, indicating that decisions based on the nomogram model may benefit childhood asthma patients (Figure 4C). The clinical impact curve revealed that the predictive power of the nomogram model was remarkable (Figure 4D).

### Two Distinct m6A Patterns Identified by Significant m6A Regulators

**Figure 5:**
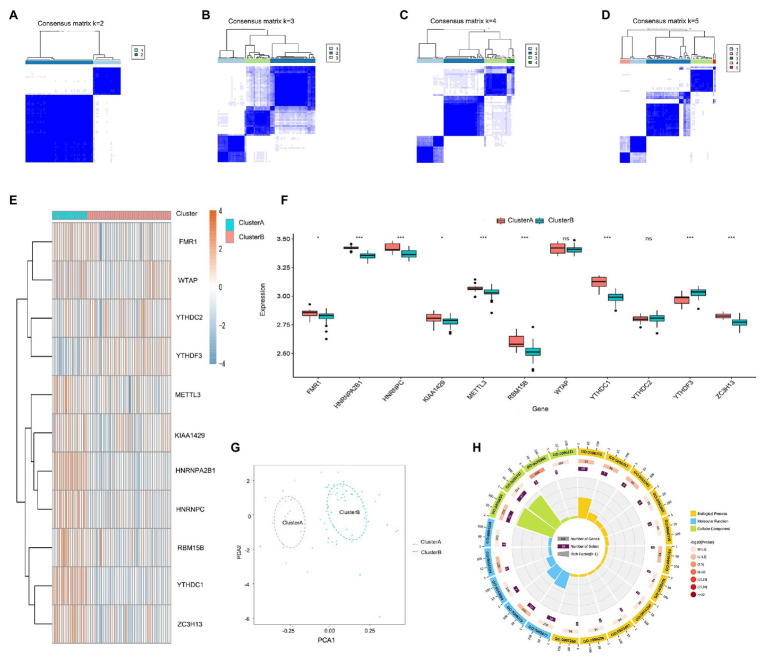
Consensus clustering of the 11 significant RNA N6-methyladenosine (m6A) regulators in childhood asthma. **(A-D)** Consensus matrices of the 11 significant m6A regulators for *k* = 2–5. **(E)** Expression heat map of the 11 significant m6A regulators in clusterA and clusterB. **(F)** Differential expression histogram of the 11 significant m6A regulators in clusterA and clusterB. **(G)** Principal component analysis for the expression profiles of the 10 significant m6A regulators that shows a remarkable difference in transcriptomes between the two m6A patterns. **(H)** Gene ontology analysis that explores the potential mechanism underlying the effect of the 119 m6A-related differentially expressed genes (DEGs) on the occurrence and development of childhood asthma. ^*^p < 0.05, ^**^p < 0.01, and ^***^p < 0.001.

The consensus clustering method was used to identify distinct m6A patterns based on the 11 significant m6A regulators using the “ConsensusClusterPlus” package in R software, and two m6A patterns (clusterA and clusterB) were identified (Figures 5A–D). ClusterA contained 19 cases, and clusterB contained 46 cases. Then, the heat map and histogram were plotted to illustrate the differential expression levels of the 10 significant m6A regulators between the two clusters. YTHDC1, HNRNPC, FMR1, HNRNPA2B1, KIAA1429, METTL3, RBM15B, and ZC3H13 displayed higher expression levels in clusterA than in clusterB while YTHDF3 showed the opposite. WTAP and YTHDC2 demonstrated no significant differences between clusterA and clusterB (Figures 5E,F). PCA indicated that the 10 significant m6A regulators could completely distinguish the two m6A patterns (Figure 5G). A total of 119 m6A-related DEGs were selected between the two m6A patterns. To understand the possible mechanism of these DEGs in childhood asthma, GO functional enrichment analysis was applied, and the results were visualized with an enrichment circle diagram (Figure 5H). We found that the genes were mainly enriched in GO:0070382, GO:0005634, GO:0005721, and GO:0005737, all of which were related to cell proliferation.

**Figure 6:**
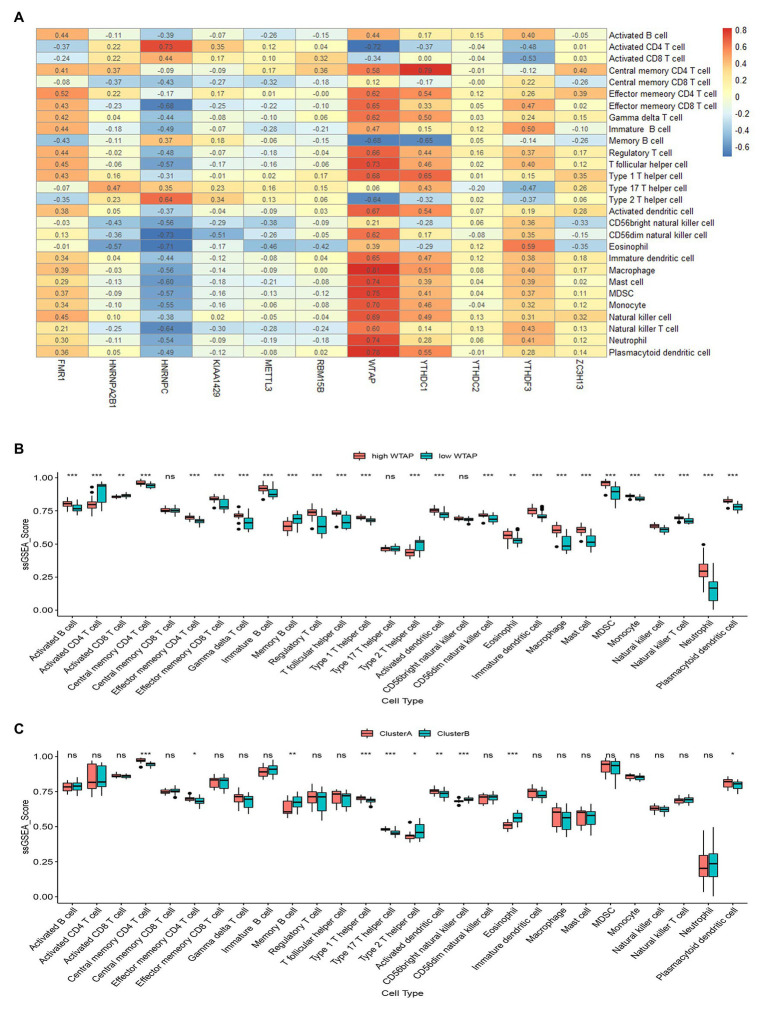
Single sample gene set enrichment analysis. **(A)** Correlation between infiltrating immune cells and the 11 significant RNA N6-methyladenosine regulators. **(B)** Difference in the abundance of infiltrating immune cells between high and low Wilm’s tumor 1-associated protein expression groups. **(C)** Differential immune cell infiltration between clusterA and clusterB. ^*^p < 0.05, ^**^p < 0.01, and ^***^p < 0.001.

We then applied ssGSEA to calculate the abundance of immune cells in asthmatic samples and evaluated the correlation between the 11 significant m6A regulators and immune cells. We found that WTAP, an m6A methyltransferase, had positive correlations with numerous immune cells (Figure 6A). We explored the differential immune cell infiltration between patients with high WTAP expression and low WTAP expression. The results indicated that patients with high WTAP expression had increased immune cell infiltration compared to patients with low WTAP expression (Figure 6B). Finally, we analyzed the differential immune cell infiltration between the two m6A patterns. We found that clusterA was linked to Th1-dominant immunity while clusterB was linked to Th2-dominant immunity, which suggested that clusterB may be related to allergic asthma (Figure 6C).

### Identification of Two Distinct m6A Gene Patterns and Generation of the m6A Gene Signature

**Figure 7:**
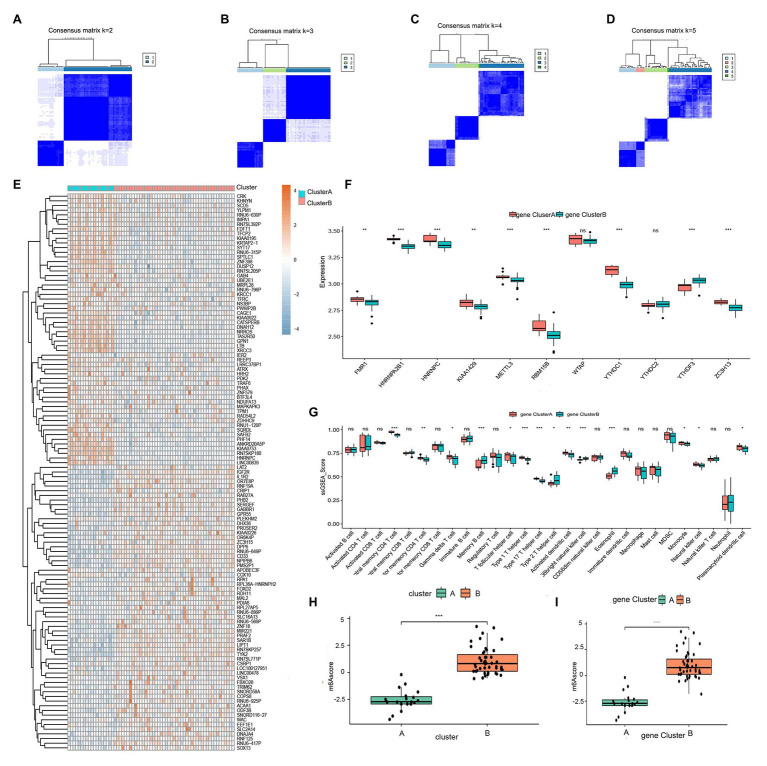
Consensus clustering of the 119 RNA N6-methyladenosine (m6A)-related DEGs in childhood asthma. **(A-D)** Consensus matrices of the 119 m6A-related DEGs for *k* = 2–5. **(E)** Expression heat map of the 119 m6A-related DEGs in gene clusterA and gene clusterB. **(F)** Differential expression histogram of the 11 significant m6A regulators in gene clusterA and gene clusterB. **(G)** Differential immune cell infiltration between gene clusterA and gene clusterB. **(H)** Differences in m6A score between clusterA and clusterB. **(I)** Differences in m6A score between gene clusterA and gene clusterB. ^*^p < 0.05, ^**^p < 0.01, and ^***^p < 0.001.

**Figure 8:**
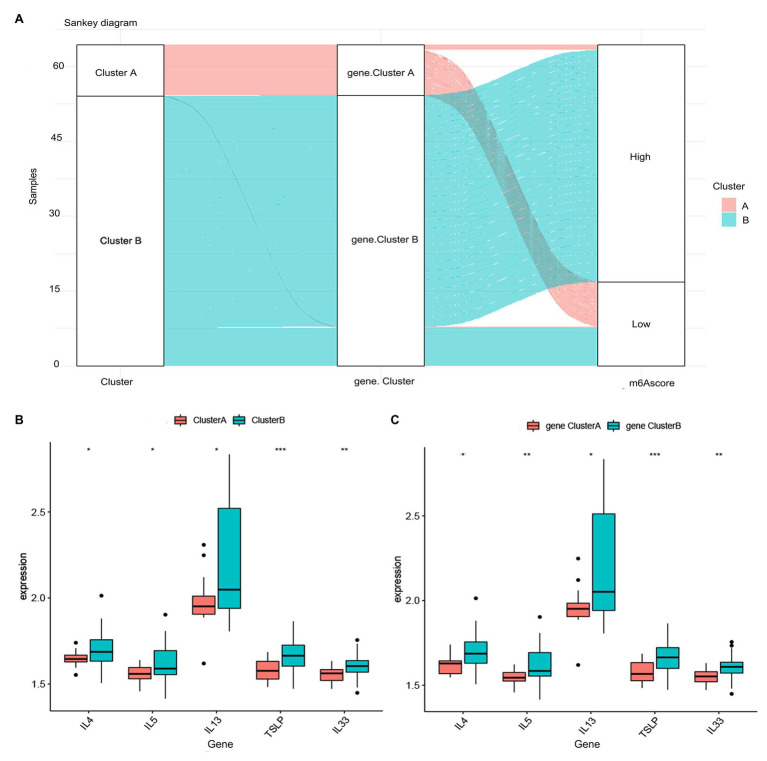
Role of RNA N6-methyladenosine (m6A) patterns in distinguishing allergic asthma. **(A)** Sankey diagram showing the relationship between m6A patterns, m6A gene patterns, and m6A scores. **(B)** Differential expression levels of thymic stromal lymphopoietin (TSLP), interleukin (IL)-33, IL-4, IL-5, and IL-13 between clusterA and clusterB. **(C)** Differential expression levels of TSLP, IL-33, IL-4, IL-5, and IL-13 between gene clusterA and gene clusterB. ^*^p < 0.05, ^**^p < 0.01, and ^***^p < 0.001.

To further validate the m6A patterns, the consensus clustering method was used to divide the asthmatic patients into different genomic subtypes based on the 119 m6A-related DEGs. We found that two distinct m6A gene patterns were present (gene clusterA and gene clusterB), which was consistent with the grouping of m6A patterns (Figures 7A–D). The expression levels of the 119 m6A-related DEGs in gene clusterA and gene clusterB are shown in Figure 7E. Figures 7F,G indicate that the differential expression levels of the 11 significant m6A regulators and immune cell infiltration between gene clusterA and gene clusterB were also similar to those in the m6A patterns. This again validates the accuracy of our grouping by the consensus clustering method. To quantify the m6A patterns, we utilized PCA algorithms to calculate the m6A score for each sample. We then compared the m6A score between the two distinct m6A patterns or m6A gene patterns. The results showed that the m6A score in clusterB or gene clusterB was higher than that in clusterA or gene clusterA (Figures 7H,I). The relationship between m6A patterns, m6A gene patterns, and m6A scores was visualized in a Sankey diagram (Figure 8A).

### Role of m6A Patterns in Distinguishing Allergic Asthma

To further reveal the relationship between m6A patterns and allergic asthma, we investigated the correlation between m6A patterns and thymic stromal lymphopoietin (TSLP), interleukin (IL)-33, IL-4, IL-5, and IL-13. The results showed that the expression levels of TSLP, IL-33, IL-4, IL-5, and IL-13 were higher in clusterB or gene clusterB than those in clusterA or gene clusterA, which suggested that clusterB or gene clusterB is highly linked to allergic asthma characterized by the Th2 immune response (Figures 8B,C).

## Discussion

Bronchial asthma is a common heterogeneous disease with respiratory system symptoms in infants (Allen, 2020). Increasing evidence has shown that m6A regulators are involved in many biological processes (Pinello et al., 2018; Tong et al., 2018a). However, the function of m6A regulators in childhood asthma remains unknown. The aim of our research was to investigate the role of m6A regulators in childhood asthma.

We first identified 11 significant m6A regulators among 21 m6A regulators through differential expression analysis between non-asthmatic and asthmatic patients. An RF model was established to select five candidate m6A regulators (FMR1, KIAA1429, WTAP, YTHDC2, and ZC3H13) from the 21 m6A regulators to predict the occurrence of childhood asthma. However, we were unable to validate our model in an independent dataset due to the lack of dataset with m6A regulators in the public database. A nomogram model based on the five candidate m6A regulators was constructed, and the DCA curve indicated that decisions based on the nomogram model may benefit childhood asthma patients. Fragile X mental retardation protein (FMRP), an RNA binding protein encoded by the FMR1 gene, is highly expressed in brain neurons and can regulate the transcription and translation of synaptic related genes. It was found that FMRP could bind to the m6A site of mRNA and maintain mRNA stability (Zhang et al., 2018). WTAP is a ubiquitous nuclear protein named after its specific binding to the Wilm’s tumor 1 protein (Little et al., 2000). WTAP plays an important role in a variety of physiological processes in cells, including binding with the 3' untranslated region of mRNA to improve mRNA stability (Horiuchi et al., 2006). As an important part of the MTC, WTAP can promote the formation of m6A (Zhang et al., 2016). In addition, WTAP is involved in cell cycle regulation and the selective splicing of mRNA (Horiuchi et al., 2013; Haussmann et al., 2016). KIAA1429 is a high molecular weight protein that may be used as a scaffold for the MTC. Through its N-KIAA1429 domain, KIAA1429 acts as a bridge between the catalytic core component of METTL3/METTL14/WTAP and the RNA substrate, thus affecting the installation of m6A at specific sites (He et al., 2019). YTHDC2 is a protein from the DExD/H box RNA helicase family that plays an important role in mRNA transcription and maintenance of mRNA stability (He et al., 2020). ZC3H13 is a CCCH zinc finger protein (Zhu et al., 2019). It was reported that ZC3H13 may be a key upstream factor of nuclear factor-κB (NF-κB) that is responsible for NF-κB activation (Luo et al., 2009). Numerous studies have shown that the five candidate m6A regulators participate in the occurrence and development of tumors, including proliferation, invasion, radiotherapy resistance, and prognosis (Zalfa et al., 2017; Zhu et al., 2019; Chen et al., 2020; He et al., 2020; Xu et al., 2020). However, there are no reports on the relationship between the five candidate m6A regulators and childhood asthma. We hope that our research can provide directions for future experimental research on these m6A regulators.

At present, most researchers believe that the dysfunction of Th subsets may be an important link in the pathogenesis of allergic asthma. The Th2-dominant response caused by Th1/Th2 imbalance is one of the important immunological mechanisms of allergic asthma. When the exogenous allergen invades the body, it is recognized and absorbed by antigen-presenting cells, and then the antigen is presented to the initial T cells. The differentiation of Th cells induced by TSLP and IL-33 leads to an immune imbalance of Th2 relative to Th1 (Kamekura et al., 2012; Menzies-Gow et al., 2020). The Th2-dominant immune response is characterized by the secretion of cytokines, such as IL-4, IL-5, and IL-13. IL-4 and IL-13 promote the differentiation of B cells and the synthesis of immunoglobulin E (IgE). IL-5 can induce the chemotaxis, recruitment, differentiation, and release of active substances of eosinophils as well as cooperate with IL-4 to stimulate B cells to synthesize IgE (Alrumaihi et al., 2020; Li et al., 2020). In our research, two m6A patterns (clusterA and clusterB) were identified based on the 11 significant m6A regulators using the consensus clustering method. ClusterB was highly linked to the Th2 immune response and had higher expression levels of TSLP, IL-33, IL-4, IL-5, and IL-13, which indicated that clusterB may be related to allergic asthma. We then confirmed the reliability of the above results in m6A gene patterns based on the 119 m6A-related DEGs. Finally, to quantify the m6A patterns, we utilized PCA algorithms to calculate the m6A score for each sample. We found that the m6A score in clusterB or gene clusterB was higher than that in clusterA or gene clusterA.

## Conclusion

In conclusion, the current study selected five candidate m6A regulators and established a nomogram model that accurately predicts the prevalence of childhood asthma. Based on the 11 significant m6A regulators, we further identified two m6A patterns, one of which (clusterB) may be related to allergic asthma.

## Data Availability Statement

The datasets presented in this study can be found in online repositories. The names of the repository/repositories and accession number(s) can be found in the article/supplementary material.

## Author Contributions

BD, FS, XC, CL, HL, and YS conceived and designed the study. BD, FS, and YS developed the methodology. BD, FS, XC, and YS analyzed and interpreted the data. BD, HL, and YS wrote, reviewed, and/or revised the manuscript. All authors contributed to the article and approved the submitted version.

### Conflict of Interest

The authors declare that the research was conducted in the absence of any commercial or financial relationships that could be construed as a potential conflict of interest.

## Footnotes

^1^http://www.ncbi.nlm.nih.gov/geo/
